# Progress in Medicine

**Published:** 1900-03-17

**Authors:** 


### Progress in Medicine.

### DISEASES OF THE NERVOUS SYSTEM.

**Headaches.**—In opening a discussion on headaches and their treatment at the British Association,[1] Lauder Brunton said that the first method of treatment is to try to supply the brain with healthy blood—one method of doing this is by a blue pill and black draught. The next thing is to give something which will have the power of counteracting toxins or of producing elimination from the liver. For this nothing is so good as a mixture of salicylate of sodium and bromide of potassium, 10-30 grains of each, taken at night. If necessary this may be given additionally two or three times during the day. The salicylate is apt to produce a certain feeling of depression and weakness, and to counteract this half a drachm of aromatic spirit of ammonia may be added to the medicine. If the patient is taking this regularly a little iron should be added to prevent the salicylate from producing anæmia.

There is another large class of drugs which are used for relieving headache—antipyrin, nux vomica, caffein, and phenacetin; they relieve the pain unless it be too severe. They ought, therefore, to be given before that happens. If given at the height of the pain, they may sometimes even make it worse. In cases where the pain is so severe that these substances seem to make it worse, the only drug which will give relief is morphia, given subcutaneously. In some cases where there is continued pain in the head lasting for a length of time, cannabis indica seems to help; 10 minims of the tincture three times a day may be given, and cautiously increased. A large initial dose of this drug is apt to bring on maniacal excitement. For the relief of headache occurring through inflammation of the periosteum from gouty, rheumatic, or syphilitic irritation, iodide of potassium is very useful.

Grant said that nasal disease may often be the unsuspected cause of headache, the most common form giving rise to this being adenoid vegetations in the naso-pharynx. Hypertrophy of the middle turbinated body, and inflammatory affections of the accessory sinuses are also common causes. Symptoms, indicating nasal disease, occurring in association with headache should lead us to suspect such an origin of the headache—such as nasal obstruction, discharges, whether mucous or purulent, fœtor, attacks of sneezing, and epistaxis. Rhinoscopy may reveal the cause of the nasal symptoms, and if the application of cocaine should afford marked relief, the impression that the local condition is the cause of the headache is almost conclusively demonstrated.

Campbell said that only in a certain proportion of cases could migraine be removed by a vegetarian diet and salicylates—others continued unabated. Large doses of iodides did much good in headaches due to organic causes. Neurasthenic headache, so common at the climacteric, often continued after every source of peripheral irritation had been removed, and every remedy had been given. In such cases a suitable climate was of very great use.

Herschell said that toxins might not merely be admitted into the general circulation through defective action of the liver, but might be produced in greater amount than normally. This was often the case in dilatation of the stomach due to atony. The only certain sign of this condition of the stomach was the finding of food residues when the stomach should be empty, but splashing below the umbilicus was a strong presumption. Such headaches could be cured by washing out the stomach. Chronic fæcal retention in the sigmoid flexure was another common cause of chronic headache.

Thomson said that headache, though paroxysmal, might be due to some abiding cause; thus a patient suffered from headache at each menstrual period for over 12 years; after evacuation of a maxillary empyema, the headache ceased.

Draffin said that men who work in extreme heat, *e.g.*, in ironworks, often develop headaches on coming into the cold; these probably arose through pressure on the vessels, for they would be relieved by the man resuming work in a hot atmosphere. A combination of antifebrin and caffein would generally relieve such a headache.

**Meningitis.**—In the course of a most practical paper on meningitis, Barr[2] says, in discussing the question of diagnosis, that the presence of headache is often overlooked because the patient is not shouting, "Oh! my head"; he is often too ill to complain. Vomiting, too, is often not an obtrusive feature. One of the earliest, most characteristic, and most persistent symptoms, especially in meningitis of the convexity, is general hyperalgesia; so that if a limb be firmly grasped the patient winces. There is also intolerance of light and sound. Among the early signs are moderate pyrexia, delirium, stupor, occasionally general convulsions, irregularity of pupils, and retraction of the head. The nature of the disease may often be surmised from the cause. Purulent meningitis is associated with middle-ear disease, necrosis of bone, abscess, septicæmia, and frequently cerebro-spinal meningitis. Simple meningitis may be connected with pneumonia, rheumatism, or some of the specific diseases. Tuberculous meningitis is most common in children under 10 years, though it may occur especially as a complication of phthisis in adult life.

There should be no difficulty in distinguishing meningitis from typhoid fever; in the latter disease the patient is dull, apathetic, and listless, and, although there may be violent headache and later on marked delirium, yet the patient is not specially sensitive to external impressions of light and sound, and there is no general hyperalgesia. In meningitis this is present, and the patient is irritable and restless, and the tendon jerks are generally pronounced. At a later stage of the disease these signs may disappear. In typhoid fever the deep reflexes are only exaggerated in severe cases and at a later stage of the disease. Widal's test does not give positive results before the fifth day of a typhoid fever, and the diagnosis should be made before then. Pneumonia of the apex may be mistaken for meningitis, but examination of the lungs and pulse-respiration ratio will differentiate the two affections. Influenza occasionally assumes a cerebral type, but the head symptoms soon pass off. In bilateral otitis media there may be headache, delirium, vomiting, convulsions, and vertigo, but when there is no meningitis the febrile process soon subsides, and there is no great emaciation. Lumbar puncture, though of no therapeutic value, may often give evidence of the presence of definite micro-organisms.

The treatment should be on the following lines: Rest in bed, in a quiet, darkened room; the head should be kept cold by ice, or cold water passing through Leiter's tubing. This should be continued until the general temperature has been for some time subnormal. Iodides and mercury should be given in syphilitic cases only. The new antipyretic drugs are of no use. Internal antiseptics, such as salol, may be given. To relieve cerebral excitement, sleeplessness, delirium, and general restlessness there is no drug equal to opium, freely administered. The only limit should be the quantity necessary to keep the patient constantly under the influence of the drug. In children over seven years of age one-sixth of a grain of morphia should be given hypodermically every four hours, and steadily increased until the patient is under its influence. For children about two grains a day suffice, but for adults it may be necessary to give three, four, five, or six grains daily. When there is much vomiting it is often of advantage to combine small doses of atropine with the morphine, but if there be much temperature this addition had better be omitted.

Under this little known plan of treatment Barr has had signal success in treatment; of the last 26 patients 10 only died, and three of these succumbed within 24 hours of admission.

[1] Lauder Brunton, Brit. Med. Jour., Nov. 4, 1899. [2] Barr, Brit. Med. Jour., Nov. 18, 1899.

### FEVERS.

(*Continued from page* 380.)

The commonest errors in the use of Widal's test, according to J. O. Symes,[33] are those due to insufficient dilution of the blood, which of itself may cause agglutination; and the use of an alkaline broth for the culture of the bacillus, the effect of which is that the bacillus has but slight motile power. When these and similar faults are avoided the errors do not amount to more than from 2 to 5 per cent. He gives some details of 151 cases, showing the accuracy of the test. Besides its ordinary application, the Widal reaction also enables us to tell whether a person has recently suffered from typhoid, and to distinguish between B. coli and B. typhosus when found in water.

**Scarlatina.**—W. T. Class[34] believes that the failure to find the organism of scarlet fever is due to the use of improper media for cultures. He employs a glycerine agar to which 5 per cent. of sterilised garden earth has been added. The growth is incubated for several days at 35 deg. Cent. In about two days whitish semi-transparent colonies appear, composed of diplococci without capsules or spores. In some specimens a transverse line runs through each half, so that it forms a sort of a tetrad. Each coccus is rather larger than a pus coccus. They are decolourised by Gram's method, and are found almost invariably in the scales of the epidermis and in the throats of scarlatinal patients. Swine were inoculated with pure cultures, and showed fever, a red rash, desquamation, and nephritis, and cultures from their kidneys produced the same organisms. In form they vary from very large diplococci to tetrads and small cocci,[35] which are well depicted in the illustrations of the author's paper in the *Medical Record*. Courtois[36] believes that the scarlatinal organism is a streptococcus related to that of erysipelas. He finds it in the urine of scarlatinal patients and at times in the blood, though here it undergoes some modification. H. O. Hall[37] insists on the importance of cow's milk in the conveyance of the disease. Thus he finds it is epidemic in all countries where this is a common food, especially of children, while it is unknown where they are fed on the mother's milk only. This is notably the case in China and Japan. Again, where goats and asses, milk is generally used, the disease is unknown. In India, though cow's milk is largely used by adults, children are suckled as in Japan till three, five, and six years old, and scarlatina is rare, though sometimes imported. The Japanese, who are forbidden milk for religious reasons, and suckle their children for five years, are entirely free from it, as they are indeed from rickets. The late T. W. Stickler[38] imagined that he had obtained a protective virus against scarlatina by taking mucus from the throats of patients, treating it with carbolic and injecting it in the skin of healthy persons. Unfortunately, it turned out that the true disease was reproduced uniformly. He at once ceased the treatment, but minute notes are given of the results. Thus the average duration of incubation was 32 hours; the time from the eruption to the appearance of desquamation averaged six and a half days. It seems certain from his unfortunate attempt that the nasal and pharyngeal mucus contains the infection, and that infection from this source must be carefully guarded against. At the meeting of the American Medical Association B. Gilbert[39] noted the risk of pet dogs and cats carrying the infection to healthy persons (this was undoubtedly the case in a recent outbreak at Bristol). In the same discussion Quayle advocated the use of lithia water, and the flushing the bowel with enemata to aid the kidneys; and Slagle recommended in addition cold packs with cold drinks. Garrison and Solomon referred to the value of acetanilid. Kühn[40] reports a case of scarlatina associated with tetany. A fairly typical rash was followed by desquamation, and, even before any symptoms of the fever occurred, rigidity commenced in the lower limbs and spread to the shoulders and jaws. The hands remained free, and the patient took food fairly, but the spasmodic symptoms continued more or less for six weeks.

**Malaria.**—Celli and Casagrandi[41] have investigated what substances best destroy mosquitos in their various stages. Sulphurous oxide, tobacco, chrysanthemum powders, gallol, and permanganate appear to be the most active, and especially petroleum poured on the surface of pools. Various pungent smokes may be used to destroy the adult insects in houses. The Commission which went to the West Coast discovered[42] that the Anopheles only breeds in puddles which contain algæ and no fish, and which are more or less permanent, whereas the harmless Culex frequents water vessels which are kept round houses. The German Commission in Italy[43] noted that malaria only occurred during three months of the year, and held that the new mosquitos each summer must get the infection from human beings who had suffered the previous year. If all these were thoroughly treated with quinine before the hot weather comes the disease would disappear. They thought, too, that the Culex mosquitos were carriers of malaria. Koch has confirmed Ross's account of the development of proteosoma, the parasite of birds, but adds certain stages, showing that it passes through a worm-like form during its growth.[44] Aug. Plehn[45] believes that the malaria parasites pass through an "alternate generation," for he finds certain granules in the blood which multiply there for a considerable period, and finally develop into true plasmodia, and only then produce clinical symptoms. These he regards as the primitive form of the parasite.

Bartianelli and Bignami[46] have most ably traced the development of all three species of malarial plasmodes, those of the tertian, quartan, and œstivo autumnal diseases, in their extra human cycles. They are clearly defined from one another, but they adopt as host the same species of mosquito. Each produces its own type of disease in man when the insect infected with it has access to a healthy person, or, indeed, several persons. None of them changes into another variety. Just as the crescent bodies go through their development in the mosquito into male and female forms, so they show that the bodies derived from the tertian and quartan agues pass through similar stages. The quartan, indeed, seems to remain in human blood for years, and more rarely produces the forms capable of external life, but even these have been taken up by mosquitos bred in confinement where they could not be infected elsewhere, and have been transferred to fresh human subjects. Moreover, the insects in the winter are free from active infection, and a study of the cases shows that in early summer they become attacked from biting persons who suffered from malaria the previous year, after which a rapid spread of the disease takes place among the population. They found the Anopheles in great numbers in the stagnant dykes near Ostia, where the great drainage works have failed to get rid of the malaria, probably because these open drains themselves afford a good breeding-place for the mosquitos. With regard to Koch's claim to have found the germs in a Culex mosquito, Berkeley[47] remarks that he offers no evidence to show that they are not the proteosoma of birds, while Ross, after dissecting "quite a thousand specimens" which had fed on malarial blood, found none, except one which he believed to be an avian parasite. Hence he regards the theory that the Culex, as well as the Anopheles, may convey malaria, as improbable.

[33] Hospital, Dec. 9. [34] Brit. Med. Jour., Nov. 25. [35] Med. Rec., Sept. 2, and Oct. 7. [36] Brit. Med. Jour., Dec. 30. [37] Med. Rec., Nov. 11. [38] Med. Rec., Sept. 9. [39] Brit. Med. Jour., July 15. [40] Med. Rec., Nov. 11. [41] Brit. Med. Jour., Sept. 9. [42] Brit. Med. Jour., Oct. 14. [43] Brit. Med. Jour., Sept. 23. [44] Brit. Med. Jour., Oct. 14. [45] Jour. Trop. Med., Dec. [46] Lancet, Jan. 13. [47] Med. Rec., Dec. 23.

